# Editorial: Relevance in mind

**DOI:** 10.3389/fpsyg.2023.1338336

**Published:** 2023-11-30

**Authors:** Tim Wharton, Kate Scott, Didier Maillat, Caroline Jagoe

**Affiliations:** ^1^School of Humanities and Social Science, University of Brighton, Brighton, United Kingdom; ^2^School of Design, Kingston University, Kingston upon Thames, United Kingdom; ^3^Department of English, Université de Fribourg, Fribourg, Switzerland; ^4^School of Linguistic, Speech and Communication Science, Trinity College Dublin, Dublin, Ireland

**Keywords:** relevance, pragmatics, relevance theory, cognition, communication

This *Frontiers* Research Topic is an attempt to push the envelope of relevance theory with particular attention to its implications for psychology and cognitive science and to disseminate the theory more widely, encouraging others to engage with the theory and better understand its capacity to broaden and deepen our understanding of all aspects of communication and cognition. With that in mind, the contributions to “*Relevance in Mind”* address one of the following three themes:

1. Relevance theory in psychology and cognitive science
2. Relevance theory, social communication, and social cognition
3. Relevance theory: extending the boundaries from within

The papers are grouped according to the theme they most closely fit, while recognizing that these themes are not mutually exclusive.

## Theme 1: Relevance theory in psychology and cognitive science

In *Intonational production as a window into children's early pragmatic competence*, Helganger and Falkum investigate what the production of the Norwegian “Polarity Focus” intonation pattern by preschool children reveals about their early pragmatic development. The mastery of this pattern, they claim, can be seen as an early linguistic manifestation of relevance-driven cognitive abilities for the attribution of thoughts and epistemic vigilance toward propositional content. Noveck et al.'s *Taking stock of an idiom's background assumptions* argues that since relevance theory has tended to focus on the interpretation of metaphor and irony, there is a great deal of work to be done on the interpretation of idioms. They argue for a relevance theory approach in which idioms are explained through the fact that they activate presuppositional information. The new approach is confirmed through a pilot experiment.

In *Strength is relevant: experimental evidence of strength as a marker of commitment*, Boulat and Maillat explore the notion of “strength”, one of the relevance-theoretic organizing principles responsible for ordering contextual assumptions. They argue for a theoretical notion in which strength is regarded as a marker of commitment, and—more generally—of the epistemic value of an utterance. This claim is supported through a set of new experiments in which levels of strength are manipulated and, in turn, shown to correlate with accuracy in a recollection task. Their results support their model and its implications are discussed. Ronderos and Falkum's *Suppression of literal meaning in single and extended metaphors* tests recent theoretical claims made by Carston on the differences between the processing of single and extended metaphors. Their work builds on claims that processing single metaphors involves suppressing features related exclusively to the literal meaning. Their goal is to investigate whether suppression is also involved in the comprehension of extended metaphors, or whether—as Carston suggests—the literal meaning “lingers”, thereby leading to the continued activation of such features. They suggest their results lend support for Carston's view.

## Theme 2: Relevance theory, social communication, and social cognition

Mari and Müller's paper *Social cognition and relevance* explores the impact of social cognition on the processing of linguistic information, demonstrating how gender and nationality-related stereotypes guide the relevance-based processing of definite and indefinite descriptions. Results show that information contradicting nationality stereotypes costs significantly more in terms of processing effort than information confirming stereotypes. Overall, the findings are consistent not only with research on stereotypes, but also the relevance theory claims on the relationship between effort and effects. In *Relevance theory and the social realities of communication*, Johnson considers one of the central tenets of intention-based theories of pragmatics: that the mental states of our interlocutors are altered on the basis of their recognition of our communicative intentions. She argues that this is not equally the case for all interlocutors and that according to various social factors, some bear an additional burden. By demonstrating how social factors affect the reality of the way social beings interact and communicate Johnson builds theoretical bridges between relevance theory and Fricker's work on *testimonial injustice*.

Bonalumi et al.'s *Communication and deniability: Moral and epistemic reactions to denials* looks at the potential effects of situations in which a speaker denies having meant what an audience understands them to have meant. They present experiments which explore those incentives a speaker might have to mislead their audience and the impact a speaker's denial might have on an audience's moral and epistemic assessments of what has been said. On the basis of their initial findings, they present an original analysis of how audiences react to denials which draws on the relevance theory approach to communication.

## Theme 3: Relevance theory: extending the boundaries from within

Carston's *The relevance of words and the language/communication divide* explores the idea that relevance theorists have tended to emphasize the communicative dimension of words (the construction of *ad hoc* senses, for example) at the expense of the morpho-syntactic side of language. Words, after all, are not only the building blocks of communicative exchanges. They are also the building blocks of linguistic form. The discussion suggests how the communicative side to words might interface with the computational (linguistic) one and how words effectively “straddle” the divide. It also presents evidence from populations with atypical development showing that both sides of the divide are affected differentially which suggests it is a natural one in human cognitive architecture.

Madella's *Relevance and multimodal prosody* presents the implications of analyzing contrastive stress in a multimodal context—specifically as *prosodic pointing*—for the teaching and learning of L2 prosodic pragmatics and the development of interpretive abilities in the L2 learner's mind. Her account sees contrastive stress as a tool which provides an extra cue to relevance theoretic stimulus ostension by altering the salience of one particular constituent in an utterance. In *Nutritional labelling, communication design, and relevance*, Scott adopts relevance theory notions as a means of explaining the relative effectiveness of three different nutrition labeling systems in communicating information and influencing consumer food choices. The relative success or failure of these labeling systems, Scott claims, are best explained in terms of the processing effort and inferential steps required from the consumer when accessing relevant contextual assumptions and deriving relevant implications in decision-making contexts. In other words, the success or failure of the various labeling systems is linked to their relevance in the context of interpretation.

## Author contributions

TW: Writing—original draft. KS: Writing—review & editing. DM: Writing—review & editing. CJ: Writing—review & editing.

